# Berberine Alleviate Cisplatin-Induced Peripheral Neuropathy by Modulating Inflammation Signal *via* TRPV1

**DOI:** 10.3389/fphar.2021.774795

**Published:** 2022-01-26

**Authors:** Jing Meng, Siyan Qiu, Ling Zhang, Min You, Haizhu Xing, Jing Zhu

**Affiliations:** ^1^ Jiangsu Key Laboratory for Pharmacology and Safety Evaluation of Chinese Materia Medica, Department of Pharmacy, Nanjing University of Chinese Medicine, Nanjing, China; ^2^ Jiangsu Province Key Laboratory of Anesthesia and Analgesia Application Technology, Xuzhou Medical University, Xuzhou, China; ^3^ Department of Neurology and Neuroscience, Johns Hopkins School of Medicine, Baltimore, MD, United States

**Keywords:** chemotherapy-induced peripheral neuropathy, neurodegenerative disorders, medicine plant, berberine, dorsal root ganglia

## Abstract

Chemotherapy induced peripheral neuropathy (CIPN) is a severe neurodegenerative disorder caused by chemotherapy drugs. Berberine is a natural monomer compound of Coptis chinensis, which has anti-tumor effect and can improve neuropathy through anti-inflammatory mechanisms. Transient receptor potential vanilloid (TRPV1) can sense noxious thermal and chemical stimuli, which is an important target for the study of pathological pain. In both vivo and *in vitro* CIPN models, we found that berberine alleviated peripheral neuropathy associated with dorsal root ganglia inflammation induced by cisplatin. We confirmed that berberine mediated the neuroinflammatory reaction induced by cisplatin by inhibiting the overexpression of TRPV1 and NF-κB and activating the JNK/p38 MAPK pathways in early injury, which inhibited the expression of *p*-JNK and mediated the expression of p38 MAPK/ERK in late injury *in vivo*. Moreover, genetic deletion of *TRPV1* significantly reduced the protective effects of berberine on mechanical and heat hyperalgesia in mice. In *TRPV1* knockout mice, the expression of NF-κB increased in late stage, and berberine inhibited the overexpression of NF-κB and *p*-ERK in late injury. Our results support berberine can reverse neuropathic inflammatory pain response induced by cisplatin, TRPV1 may be involved in this process.

## 1 Introduction

The platinum analogue cisplatin (cis-di-amino-di-chloro-platinum, CDDP) was the first platinum-based cytotoxic agent (Quasthoff and Hartung, 2002). Long-term use of CDDP can cause peripheral nerve injury (Staff et al., 2017), known as chemotherapy-induced peripheral neuropathy (CIPN). Platinum-based chemotherapeutics are notably toxic to neurons. Platinum leads to the dysfunction of neuronal membrane excitability and disruption of neurotransmission, induction of inflammation *via* release of proinflammatory chemokines, and alteration of voltage-gated ion channels expression (Ibrahim and Ehrlich, 2020). Platinum ion accumulation leads to DNA damage in dorsal root ganglia (DRG) of the spinal cord. And platinum also induces apoptosis in sensory neurons (Fukuda et al., 2017), resulting in chronic dose-dependent pain and sensory changes. At present, there is no specific clinic drug for treating CIPN. The main clinical treatment is analgesia, supplemented with other sedative drugs. Nonsteroidal anti-inflammatory drugs can temporarily control symptoms for some patients, but the treatment effect in most patients is not satisfactory. Although a variety of pharmacologic agents have been evaluated for the treatment of CIPN, only duloxetine (DLX) has been recommended to treat CIPN by American Society of Clinical Oncology.

CIPN is one type of chronic neuropathic pain, the expression of transient receptor potential vanilloid (TRPV1) in DRG is an important target which initiates neuron inflammation and paraesthesia. TRPV1 is widely expressed in the middle- and small-diameter neurons of DRG, as well as trigeminal ganglia (TG) (Xu et al., 2013). As a capsaicin receptor and non-selective cation channel, TRPV1 is the primary detector of chemical stimuli and thermally (>43°C) evoked pain sensations (Caterina et al., 1997; Caterina et al., 2000; Davis et al., 2000). By using the mouse model of CDDP-induced neuropathy, researchers found that treatment with CDDP results in an increase of TRPV1 mRNA level in cultured DRG neurons and the similar up-regulation of TRPV1 occurs in CDDP-treated mice (Ta et al., 2009; Ta et al., 2010). Thus, TRPV1 plays a key role in CDDP-induced thermal hyperalgesia in animal models.

In our preliminary works, we found that berberine (BBR) protects DRG nerve cells from damage by chemotherapeutic drugs. Therefore, the protective effect of BBR and its role in inflammation associated with CIPN were explored. BBR is the main active monomer in *Coptis chinensis* and a quaternary ammonium isoquinoline alkaloid, with a wide range of clinical applications (Wu et al., 2016). Recently, more indications of positive effects of BBR have been found. BBR can inhibit the expression and transcription of pro-inflammatory factors IL-1*α*, IL-1*β*, IL-8, and TNF-*α* by inhibiting JNK and TAK1/NF-κB signalling pathways (Zhang et al., 2014; Ni et al., 2021). Other studies have shown that BBR can inhibit the activity of COX-2 and exert anti-inflammatory effects through the PKC pathway, which regulates TRPV1 receptors (Zan et al., 2017). In this study, we observed the protective effects of BBR on neuroinflammatory injury induced by chemotherapeutic drugs and BBR treatment of CIPN does not affect the anti-tumour effect of chemotherapy drugs.

In summary, using CDDP peripheral nerve injury animal and cell models *via TRPV1* gene knockout, we observed changes in inflammatory factors and signals after chronic nerve injury and found the neuroprotective effects of BBR on CIPN.

## 2 Materials and Methods

### 2.1 Experimental Animals

All procedures involving animals were performed in accordance with the ethical guidelines established by the International Association for the Study of Pain. This study was approved by the Animal Care and Use Committee of Nanjing University of Chinese Medicine (Nanjing, China). Male C57BL/6 [wild-type (WT)] and *TRPV1* ^−/−^ mice weighing 20–22 g was used for these experiments. C57BL/6 mice were purchased from Qinglongshan Animal Centre, Nanjing, China. *TRPV1* knockout mice were obtained from Johns Hopkins University (Baltimore, MD, United States). After backcrossing *TRPV1* ^−/−^ mice with WT mice for six generations, *TRPV1* ^−/−^ mice were mated with each other to generate null mutant offspring for use in the present study. All animals were held in a humidity- and temperature-controlled environment and maintained in a 12:12 h light and dark cycle with free access to food and water. All behavioural experiments were performed by an individual blinded to the treatment groups.

### 2.2 Cisplatin Induced Peripheral Neuropathy Model and Drug Administration

Mice were randomly divided into six groups (*n* = 8 per group). CDDP was dissolved in 0.9% sterile saline to obtain a final concentration of 0.3 mg/ml. CDDP (3 mg/kg) was injected intraperitoneally (i.p.) 8 times (Ta et al., 2009; Carozzi et al., 2014). Normal control mice were injected with sterile saline, consistent with the quantities injected into mice in the drug treated groups. Low (60 mg/kg), medium (90 mg/kg), and high (120 mg/kg) concentrations of BBR were administered orally daily in the first 2 weeks and every other day after 2 weeks. Mice in the duloxetine positive control group were intraperitoneally injected with duloxetine (20 mg/kg, prepared with normal saline). Behavioural tests were performed weekly. The thermal pain threshold in mice were measured at 1, 2, 3, 4, and 8 h after the first administration of CDDP.

### 2.3 Behavioral Assay

#### 2.3.1 The Radiant Heat Assay

The thermal radiation tester (37370-001, Ugo Basile, Italy) was used to measure heat hyperalgesia. Mice were placed in a 3 mm thick plexiglass box for 30 min, the light source was directed at the skin of the middle and posterior part of the hind foot of mice, the paw withdrawal latency (PWL) of mice were recorded.

#### 2.3.2 Hot Plate Assay

The hot plate (35150-001, Ugo Basile, Italy) was used to determine heat thresholds of mice. Mice were placed on a metal surface maintained at a constant temperature (55 ± 0.5°C). The time from feet touching the hot plate to responding of the hind foot (paw withdrawal or licking, stamping) were recorded. A cut-off time of 60 s was applied to prevent pelma pain.

#### 2.3.3 Cold Plate Assay

The cold plate (35150-001, Ugo Basile, Italy) was used to determine heat thresholds of mice. Mice were placed on a metal surface maintained at a constant temperature (4 ± 0.5°C). The time from feet touching the cold plate to responding of the hind foot (paw withdrawal or licking, stamping) were recorded. A cut-off time of 60 s was applied to prevent pelma pain.

#### 2.3.4 Tail Flick Test

Half of the tail of each mouse was placed in a 48°C constant temperature water bath. The tail flick latency (TFL) of mice were determined according to the time of immersion into water to tail flick out of the water. A cut-off time of 40 s was applied to prevent tail pain.

#### 2.3.5 Mechanical Allodynia

Mechanical allodynia was evaluated using von Frey filaments (37450-001, Ugo Basile, Italy) according to a previously reported method. Mice were placed individually in small cages with a mesh floor for 30 min. A monofilament was applied perpendicularly to the plantar surface of the hind paw, gradually increase the intensity of the stimulus. The time from the beginning of stimulation to raising, licking, or jumping was considered the paw withdrawal latency (PWL, s) time, and the stimulus intensity was the paw withdrawal threshold (PWH, g). Measurements were repeated three times at 5-min intervals, and the mean value was calculated.

### 2.4 Western Blot

DRG tissues were collected from mice and later disrupted in RIPA mixed with PMSF and protease inhibitor cocktail (1×) on ice. Total protein (20 μg) was separated by 10% SDS polyacrylamide gels and then transferred to PVDF membranes (Bio-Rad). After the membranes were blocked for 1 h at room temperature in Tris-buffered saline Tween-20 (TBST) containing 5% skim milk, followed by incubation with primary antibodies specific for p38MAPK (1:4000, CST, Danvers, MA, United States), *p*-p38MAPK (1:4000, CST), ERK1/2 (1:4000, Abcam, MA, United States), *p*-ERK1/2 (1:4000, CST), JNK (1:3000, Abcam), *p*-JNK (1:3000, Abcam), NF-κB (1:4000, Abcam), TRPV1 (1:2000, Neuromics, INA), and GAPDH (1:8000, CST) at 4°C overnight. After being washed with TBST three times, the membranes were incubated with secondary antibodies (1:10,000, Proteintech, WH, CHN) for 1 h at room temperature. After being washed with TBST three times, the antibodies were detected with ECL reagents (Tanon, SH, CHN). Immunoreactivity was detected by chemiluminescence. The results were quantified using ImageJ software.

### 2.5 Total RNA Extraction and Polymerase Chain Reaction (PCR)

Total RNA was extracted from DRG tissues using Trizol/chloroform reagent (Thermo-Scientific, United States). RT-PCR was proceeded according to a First Strand cDNA Synthesis Kit (TOYOBO, Japan) and Polymerase Chain Reactor (ABI, United States). Q-PCR was using TransStart Top Green qPCR SuperMix (TransGen Biotech, Beijing, CHN) on a Real-Time PCR System (Applied Biosystems, Foster City, CA, United States). The GAPDH was used as an endogenous reference, its forward primer was TTC CTA CCC CCA ATG TAT CCG, and the reverse primer was CAT GAG GTC CAC CAC CCT GTT. The mRNA expression levels of *NF-κB* and *TRPV1* in DRG tissues were determined using quantitative RT-PCR. The NF-κB forward primer was CTG GTG CAT TCT GAC CTT GC, and the reverse primer was GGT CCA TCT CCT TGG TCT GC. The *TRPV1* forward primer was AAG AGC AAG AAG CGC CTG AC, and the reverse primer was GCA GAG CAA TGG TGT CGT TC. The relative quantification of real-time RT-PCR products was performed using the 2^−ΔΔCT^ method (Livak and Schmittgen, 2001).

### 2.6 Immunofluorescence

DRG tissues and primary DRG neurons were fixed with 4% paraformaldehyde for 20 min and washed with phosphate-buffered saline (PBS). The sections were immersed in 0.4% Triton X-100 for 15 min and blocked in 5% BSA for 1 h at room temperature. The sections were then stained with *β*Ⅲ-tubulin mouse monoclonal antibody (1:500, Abcam), anti-TRPV1 (VR1-C)-mouse specific antibody (1:300, Neuromics, INA), and anti-NF-κB antibody (1:500, Abcam, United States) at 4°C overnight, followed by incubation with FITC-conjugated goat anti-mouse IgG (1:200, Proteintech, CHN) for 1 h at room temperature. Sections were treated with DAPI, and immunofluorescence images were visualised using a fluorescence microscope (IX71, Olympus, Japan).

### 2.7 Enzyme-Linked Immunosorbent Assay (ELISA)

The serum levels of inflammatory factors, including nerve growth factor (NGF), NF-κB, TNF-*α*, IL-1*β*, IL-6, and IL-10, were assayed using ELISA kits (MaiBo, Nanjing, CHN). The standard curve was drawn in Excel, the regression equation and *R* values were obtained, and the concentration of each test sample was calculated.

### 2.8 Cell Culture and Treatments

Newborn rats were anaesthetised with isoflurane and then decapitated and quickly cut with surgical scissors to isolated DRG. All the above operations were performed on ice. DRG tissues were transferred to L15 (Gibco, United States) for centrifugation, and the supernatant was discarded. Collagenase I (Worthington Biochemical Corporation, United States) was added to cells for 50 min, then the foetal bovine serum (FBS, Gibco) was added to terminate digestion. The deposit was washed with L15. The DRG cells were suspended in neurobasal medium (Gibco, United States) with 10% FBS and 10 ng/ml glial cell line-derived neurotrophic factor (GDNF, Gibco, United States). After suspension, 10,000 cells/mL were counted on a blood count plate and inoculated into poly-L-lysine (Sigma, United States) coated slides or orifice plates. After 24 h of incubation in a 37°C and 5% CO_2_ incubator, the DRG neuron culture medium was exchanged for drug-containing culture medium.

CDDP was diluted in 2% FBS neurobasal culture medium to a final concentration of 3 μM. BBR was diluted to concentrations of 10 nM, 30 nM, 100 nM, 300 nM, 1 μM, 3 μM, 10 μM, and 30 μM in neurobasal medium containing 3 μM CDDP and 2% FBS. In the control group, 2% FBS-containing neuron culture medium was added. The cells were then incubated at 37°C and 5% CO_2_ incubator for 48 h. Cell viability was measured using a 3-(4,5-dimethylthiazol-2-yl)-2, 5-diphenyltetrazolium bromide (MTT) assay.

### 2.9 Data Analysis

All data are expressed as means ± standard errors of means. One-way ANOVA with correction for multiple comparisons was performed in GraphPad Prism 8. Animal behaviour data were analysed using repeated ANOVA. Statistical significance was set at *p* < 0.05.

## 3 Results

### 3.1 Berberine Protects Against CDDP-Induced Hyperalgesia

**FIGURE 1:**
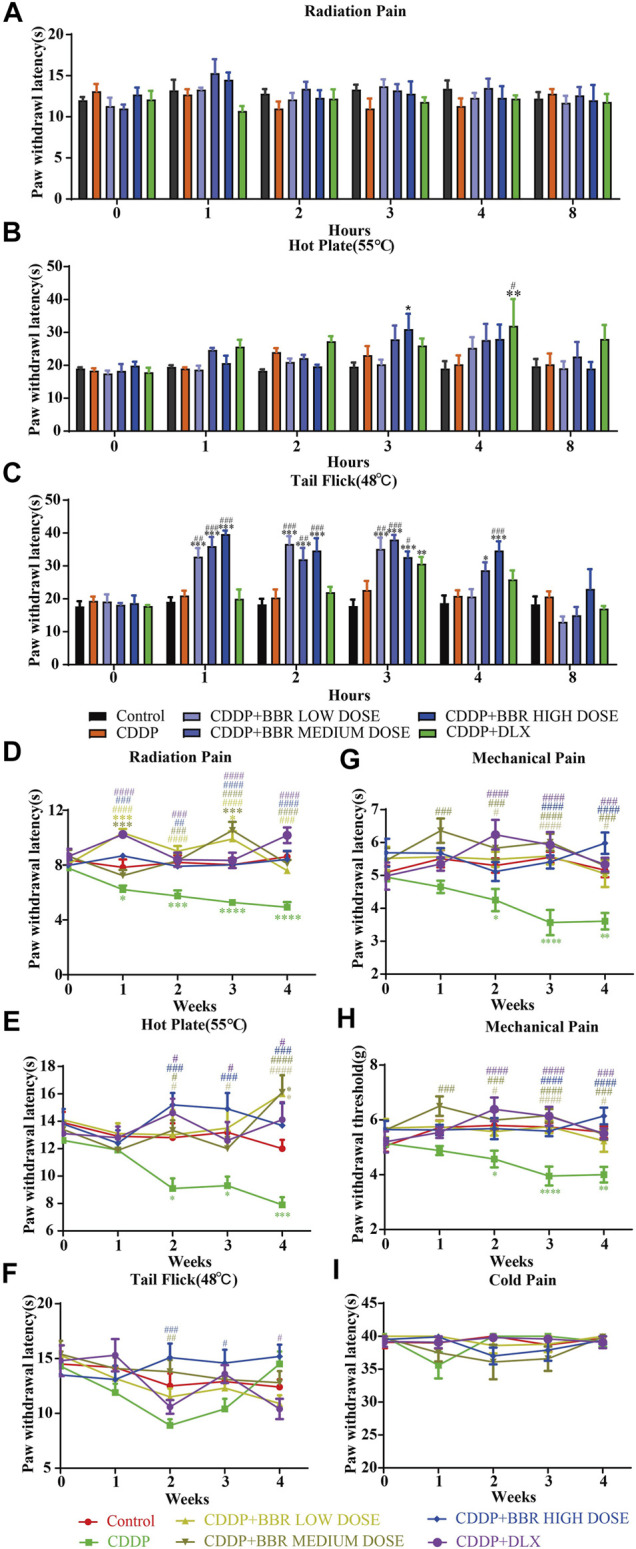
BBR increased the pain threshold and inhibited thermal hyperalgesia induced by CDDP. **(A)** The latent period of paw withdrawal induced by light radio stimulation. **(B)** The paw withdrawal latency stimulated by 55°C hot plate. **(C)** Preventive administration of BBR increased the latency of tail flick of mice for 2–3 h after immersion in a 48°C water bath. The thermal pain threshold of the pelma [**(D)**: *F* = 4.983, *p* < 0.0001] and tail [**(E)**: *F* = 1.927, *p* = 0.0007] [**(F)**: *F* = 2.086, *p* = 0.004] in CDDP mice decreased significantly after 2 weeks treatment, and the tail thermal heat pain threshold gradually increased after the third week. **(G,H)** The foot mechanical pain threshold in CDDP model mice was significantly lower than that of control [**(G)**: *F* = 1.994, *p* = 0.008]. After berberine treatment, the pain improved significantly [**(H)**: *F* = 2.206, *p* = 0.0059]. **(I)** The latency of tail responses to cold pain was no significant difference. Statistical analysis was performed using repeated ANOVA (*n* = 6–8; *p < 0.05, **p < 0.01, ***p < 0.001, ****p < 0.0001, compared with control group. ^#^ *p* < 0.05, ^##^ *p* < 0.01; ^###^ *p* < 0.001 compared with the CDDP group). CBL: CDDP + BBR low dose; CBM: CDDP + BBR medium dose; CBH: CDDP + BBR high dose.

A significant (*p* < 0.05, *p* < 0.001) development of thermal hyperalgesia was observed in the CDDP group compared to the control (Figure 1). Preventive administration of BBR significantly increased the pain threshold of mice for 2–3 h (*p* < 0.01, *p* < 0.001) (Figure 1C). Duloxetine treatment also increased the thermal heat pain threshold of mice (*p* < 0.01, *p* < 0.001) (Figure 1). The above results suggest that CDDP can induce thermal hyperalgesia in mice after a single administration for 2 h, and the pain disappears gradually after 3–4 h. All doses of BBR (low dose 60 mg/kg, medium dose 90 mg/kg, high dose 120 mg/kg) can increase the pain threshold and inhibit thermal hyperalgesia induced by CDDP. The effect of BBR was similar to that of duloxetine group.

After 1 week of administration, the thermal heat pain threshold of the pelma and tail decreased in the CDDP group mice. However, the difference from the control was not significant. Two weeks later, the thermal pain threshold of the pelma and tail in CDDP mice decreased significantly (*p* < 0.05, *p* < 0.01, *p* < 0.001) (Figures 1D–F), and the tail thermal heat pain threshold gradually increased after the third week, possibly because of severe tail injury and sensory numbness. Duloxetine only alleviated CDDP-induced pain behaviour with radiation heat and hot plate test (Figures 1D,E), but showed no significant effect on tail withdrawal latencies (Figure 1F). BBR can improve hypersensitivity induced by radiation heat and thermal stimulation at 55°C (Figures 1D,E), and BBR (90 mg/kg and 120 mg/kg) increased the tail flick threshold by the second week (*p* < 0.01*, p* < 0.001) (Figure 1F).

At the second, third, and fourth week, the foot mechanical pain threshold in CDDP model mice was significantly lower than that of control, and the mechanical pain threshold in the duloxetine group was significantly higher than that in the CDDP group (*p* < 0.001) (Figures 1G,H). Compared with the CDDP group, BBR increased the threshold of mechanical pain, and 90 mg/kg BBR was found to be the optimum concentration for this increase (Figures 1G,H). There was no significant difference in tail responses to cold pain (Figure 1I).

### 3.2 Berberine Inhibits CDDP-Induced Serum Pro-inflammatory Factors

**FIGURE 2:**
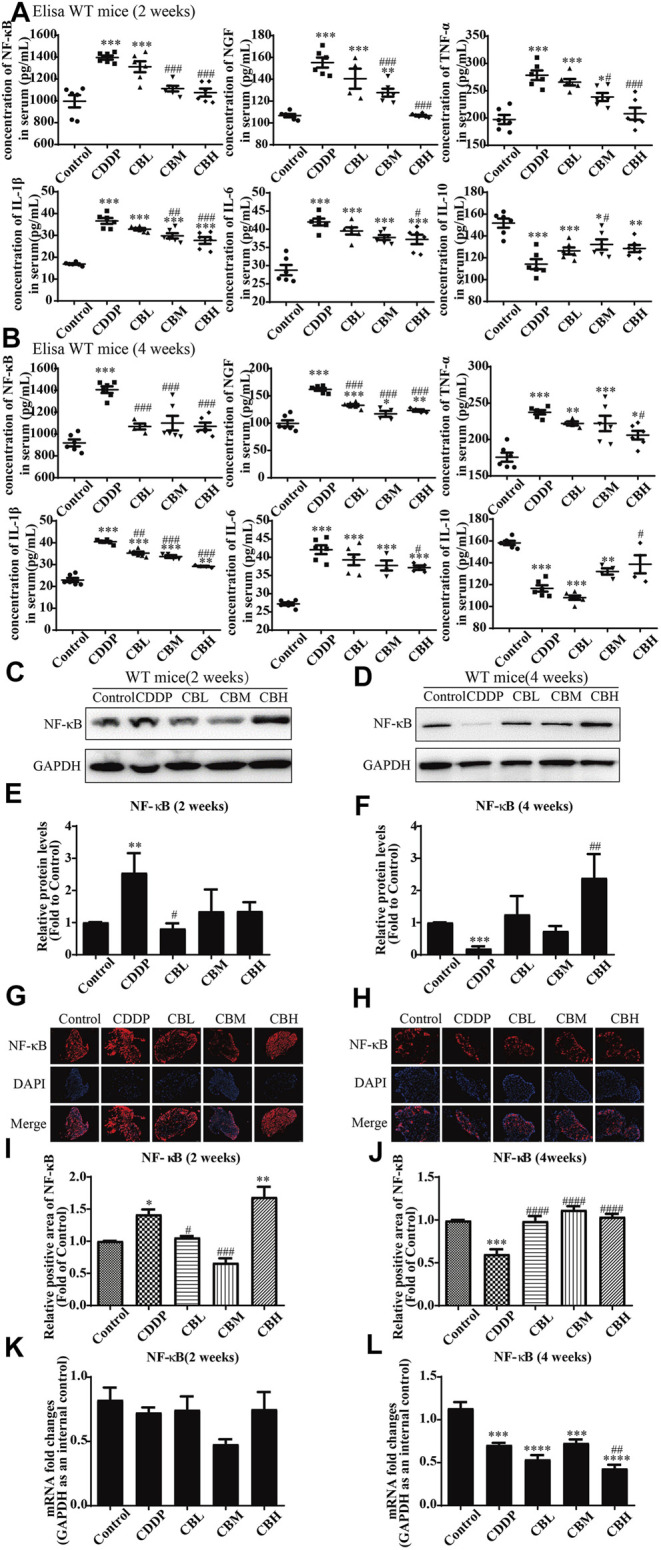
BBR reduces inflammation in serum and the overexpression of NF-κB protein in DRG in CDDP model of WT mice. **(A,B)** The effect of BBR on serum levels of inflammatory factors NF-κB, NGF, TNF-*α*, IL-1*β*, IL-6 and IL-10 were in CDDP-induced mice by ELISA. CDDP induced NF-κB (*F* = 17.81, *p* < 0.001), NGF (*F* = 28.46, *p* < 0.001), TNF-*α* (*F* = 17.34, *p* < 0.001), IL-1*β* (*F* = 49.53, *p* < 0.001), and IL-6(*F* = 20.90, *p* < 0.001) up-regulation in DRG for 2 and 4 weeks, and BBR reversed inflammatory factors up-expression in DRG. CDDP induced IL-10 under-regulation in DRG for 2 and 4 weeks, and BBR inhibited IL-10 under-regulation in DRG (*F* = 11.30, *p* < 0.001). **(C,E)** Western blot results and their quantitative data show CDDP induced NF-κB up-regulation in spinal DRG for 2 weeks, and BBR inhibited NF-κB over-expression in DRG, after 2 weeks of treatment. **(D,F)** Western blot results and their quantitative data show CDDP induced NF-κB under-regulation lasted for 4 weeks (*F* = 3.645, *p* = 0.0288). **(G,I)** Immunofluorescence results and the quantitative data show CDDP induced NF-κB up-regulation, and BBR inhibited NF-κB over-expression in DRG after 2 weeks of treatment. **(H,J)** Immunofluorescence results and their quantitative data show CDDP induced NF-κB under-regulation lasted for 4 weeks. **(K)** There was no significant difference in the expression of NF-κB mRNA after 2 weeks of BBR treatment (*p* > 0.05). **(L)** The expression of NF-κB protein and mRNA decreased at 4 weeks (*F* = 30.54, *p* < 0.0001). Statistical analysis was performed using one-way ANOVA (*p < 0.05, **p < 0.01, ***p < 0.001, ****p < 0.0001, compared with the control group; ^#^ *p* < 0.05, ^##^ *p* < 0.01, ^###^ *p* < 0.001, ^####^ *p* < 0.0001, compared with the CDDP group). CBL: CDDP + BBR low dose; CBM: CDDP + BBR medium dose; CBH: CDDP + BBR high dose.

Compared with the CDDP group, BBR 2- and 4-weeks treatment group decreased the levels of pro-inflammatory factor NF-κB, NGF, IL-1*β*, IL-6, and TNF-*α* in mice serum and increased the level of anti-inflammatory factor IL-10 (Figures 2A,B). This suggests that the anti-inflammatory effect of BBR may be mediated by the mobilisation of serum inflammatory factors.

### 3.3 Berberine Regulates Nuclear Factor NF-κB and JNK/p38MAPK/ERK Inflammatory Pathways

**FIGURE 3:**
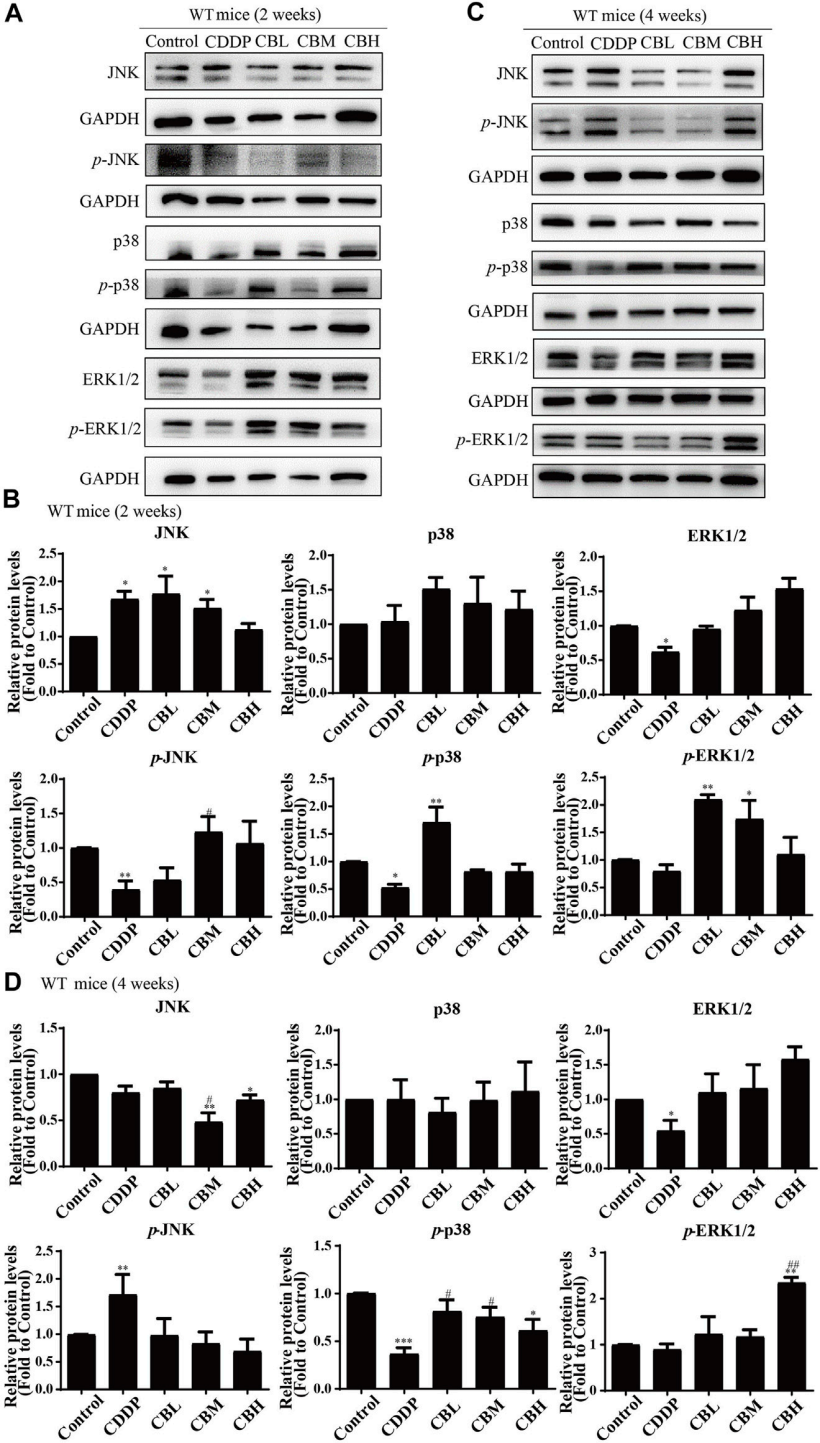
BBR regulates JNK/p38MAPK signaling pathway. **(A,B)** Western blot results and their quantitative data show CDDP induced *p*-JNK and *p*-p38 under-regulation in spinal DRG for 2 weeks, and BBR inhibited *p*-JNK and *p*-p38 under-expression in DRG. **(C,D)** Western blot results and their quantitative data show CDDP induced ERK1/2 (*F* = 2.817, *p* = 0.0838) and *p*-p38(*F* = 6.596, *p* = 0.0029) under-regulation in spinal DRG for 4 weeks, and BBR inhibited ERK1/2 and *p*-p38 under-expression in DRG. Statistical analysis was performed using one-way ANOVA (*n* = 6, *p < 0.05, **p < 0.01, ***p < 0.001, compared with the control group; ^#^ *p* < 0.05, ^##^ *p* < 0.01, ^###^ *p* < 0.001, compared with the CDDP group). CBL: CDDP + BBR low dose; CBM: CDDP + BBR medium dose; CBH: CDDP + BBR high dose.

Long-term use of CDDP causes a neuroinflammatory response and interferes with inflammatory signalling. Western blot results showed that the expression of NF-κB in DRG increased significantly after 2 weeks of CDDP use (Figures 2C,E), and the expression of NF-κB protein (Figures 2D,F) and mRNA (Figure 2L) decreased at 4 weeks (*p* < 0.001). This suggested that NF-κB participated in neurological damage induced by CDDP, but there were opposing effects at early and late stages of treatment. A high dose of BBR can reverse the expression of NF-κB induced by CDDP (Figures 2G–I). Moreover, after 2 weeks of BBR treatment, the overexpression of proteins (JNK, *p*-JNK, and p38) induced by CDDP were alleviated in the DRG (Figures 3A,B). At 4 weeks of BBR treatment, CDDP-induced DRG inflammation (Figures 3C,D) was ameliorated mainly by promoting the expression of ERK1/2 and *p*-p38 and inhibiting JNK phosphorylation. JNK/ERK/p38MAPK signalling pathways play crucial roles in BBR protection against CDDP-induced neuroinflammatory injury.

### 3.4 Effect of Berberine on TRPV1 Expression in Dorsal Root Ganglia

**FIGURE 4:**
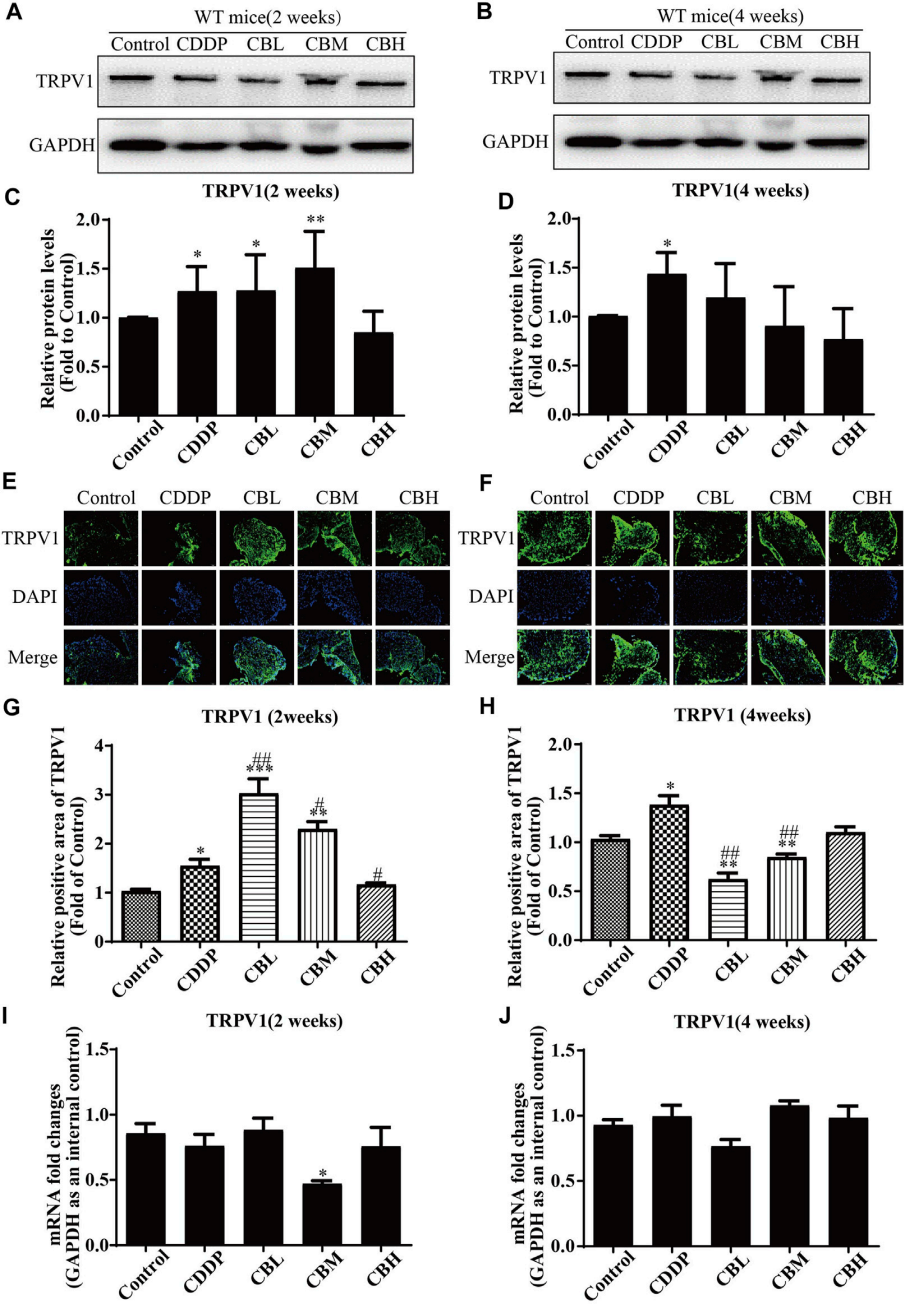
BBR inhibits up-regulation of TRPV1 protein in CDDP-induced DRG of WT mice. **(A,C)** Western blot results and their quantitative data show CDDP induced TRPV1 up-regulation in spinal DRG for 2 weeks. **(B,D)** Western blot results and their quantitative data show CDDP induced TRPV1 up-regulation lasted for 4 weeks, and BBR inhibited TRPV1 over-expression in DRG after 4 weeks of treatment. **(E,G)** Immunofluorescence results and their quantitative data show CDDP induced TRPV1 up-regulation in DRG for 2 weeks. **(F,H)** Immunofluorescence results and their quantitative data show CDDP induced TRPV1 up-regulation lasted for 4 weeks, and BBR inhibited TRPV1 over-expression in DRG after 4 weeks of treatment. Statistical analysis was performed using one-way ANOVA (*p < 0.05, **p < 0.01, ***p < 0.001, compared with the control group; ^#^ *p* < 0.05, ^##^ *p* < 0.01, ^###^ *p* < 0.001, compared with the CDDP group). CBL: CDDP + BBR low dose; CBM: CDDP + BBR medium dose; CBH: CDDP + BBR high dose.

CDDP induced TRPV1 up-regulation (Figures 4A,C) in spinal DRG for 2 weeks, and this effect lasted for 4 weeks (Figures 4B,D). BBR inhibited TRPV1 over-expression only in the high-dose treatment group by the second week. After 4 weeks of treatment, BBR inhibited TRPV1 over-expression in DRG (Figures 4B,D). The results of immunofluorescence staining of TRPV1 were consistent with those of western blotting (Figures 4E–H). There was no significant difference between *TRPV1* mRNA expression in DRG in all groups (Figures 4I,J).

### 3.5 Behavioural Changes Associated With Pain in TRPV1 ^−/−^ Mice

**FIGURE 5:**
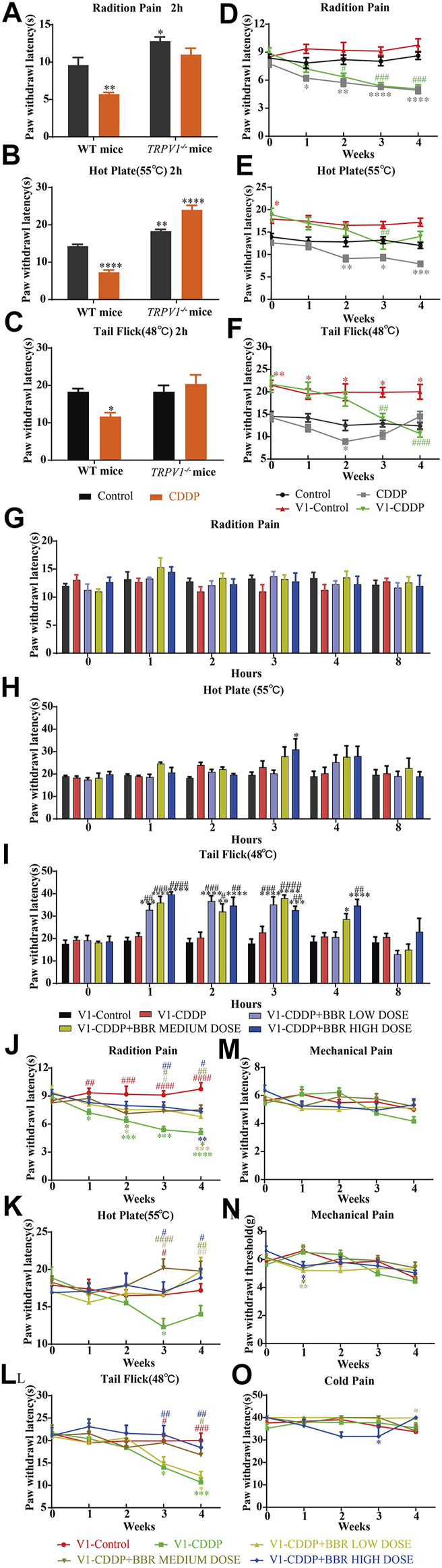
BBR increased the pain threshold and inhibited thermal hyperalgesia induced by CDDP in *TRPV1* ^*−/−*^ mice. **(A–C)** Heat pain thresholds in *TRPV1* ^*−/−*^ mice increased 2 h and 4 weeks after initial administration. *TRPV1* ^*−/−*^ mice were less sensitive to heat pain than WT mice. (*F* = 2.039; *p < 0.05; **p < 0.01, ****p < 0.0001, compared with the control group of WT mice). **(D–F)** Heat pain thresholds in *TRPV1* ^*−/−*^ mice increased 4 weeks after initial administration. *TRPV1* ^*−/−*^ mice were less sensitive to heat pain than WT mice (*F* = 1.54; *p < 0.05; **p < 0.01; ***p < 0.001, ****p < 0.0001, compared with the control group of WT mice; ^#^ *p* < 0.05, ^##^ *p* < 0.01, ^###^ *p* < 0.001, ^####^ *p* < 0.0001, V1-CDDP group compared with the V1-control group). **(G–L)** After single administration and 4 weeks, BBR effectively increased the heat pain threshold of *TRPV1* ^*−/−*^ mice (*F* = 4.527, *p* < 0.0001). **(M–O)** There was no significant change in mechanical and thermal pain thresholds in *TRPV1* ^*−/−*^ mice (*F* = 2.378, *p* = 0.0028). Statistical analysis was performed using repeated ANOVA (*n* = 6; *p < 0.05, **p < 0.01, ***p < 0.001, ****p < 0.0001, compared with the control group, ^#^ *p* < 0.05, ^##^ *p* < 0.01, ^###^ *p* < 0.001, ^####^ *p* < 0.0001, compared with the CDDP group). CBL: CDDP + BBR low dose; CBM: CDDP + BBR medium dose; CBH: CDDP + BBR high dose.

*TRPV1* knockout mice had a higher thermal heat pain threshold than WT mice. WT mice were hypersensitive to heat at 2 h after a single injection of CDDP, but *TRPV1* knockout mice were not (Figures 5A–C). After continuous injection of CDDP, a hypersensitivity to heat stimulation in WT mice was apparent at approximately 2 weeks, the *TRPV1* knockout mice were delayed to about 3 weeks (Figures 5D–F). The behavioural results suggest that CDDP-induced neurofibrillary hyperalgesia may be associated with increased expression of *TRPV1*. After the *TRPV1* gene is deleted, hyperalgesia can be controlled within 2 weeks, but other thermal hyperalgesia responses can occur after 3 weeks.

The BBR treatment groups were similar to the control group in their thermal pain thresholds on the first day of administration in *TRPV1* ^−/−^ mice (Figures 5G–I). The tail-flick latency was significantly increased at 1–3 h after BBR administration in *TRPV1* ^*−/−*^ mice (Figure 5I). This indicated that the *TRPV1* gene knockout has a synergistic effect with BBR.

After continuous administration, there was no difference in the pain threshold between the BBR group and the control group in *TRPV1* gene knockout mice. BBR led to remission of CDDP-induced thermal hyperalgesia after 3 weeks (Figures 5J,K,L). There was no difference in the responses of the mice in different groups to mechanical and cold stimuli (Figures 5M–O). This suggested that BBR treatment in the late stage of CIPN may involve other mechanisms besides that associated with TRPV1.

### 3.6 Effect of Inflammatory Factors in the Sera of TRPV1^−/−^ Mice

**FIGURE 6:**
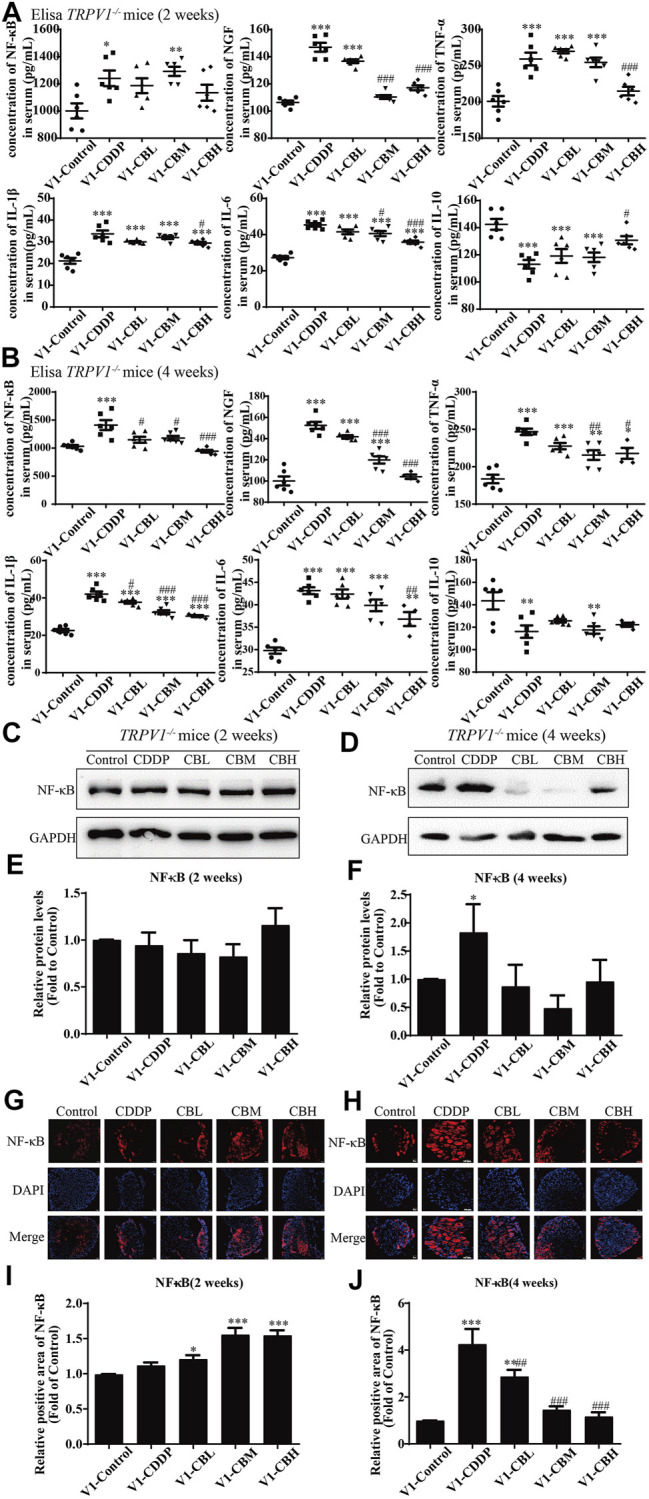
BBR reduces inflammation in mice and inhibits the overexpression of NF-κB in *TRPV1* ^*−/−*^ mice induced by CDDP. **(A,B)** Expression of inflammatory cytokines NF-κB, NGF, IL-1*β*, IL-6, TNF-*α* and IL-10 in serum were determined by ELISA (*n* = 4–6). BBR can down-regulate the expression of pro-inflammatory cytokines NGF, TNF-*α*, IL-1*β*, IL-6 and up-regulate the expression of anti-inflammatory cytokines IL-10 in the serum of *TRPV1* ^*−/−*^ mice. **(C–F)** Western blot showed the levels of NF-κB in the DRGs of *TRPV1* ^*−/−*^ mice. **(G–J)** Immunofluorescence staining showed NF-κB relative abundance in DRG of *TRPV1* ^*−/−*^ mice. Section Thickness = 10 mm, Scale bar = 50 μm. **(C,E,G,I)** There was no difference in the protein expression of NF-κB in DRG of mice in each group at the second week after *TRPV1* knockout. **(D,F,H,J)** At the fourth week after *TRPV1* knockout, the expression of NF-κB was significantly increased in the CDDP group, while BBR treatment could inhibit its overexpression (*F* = 17.24, *p* = 0.0002). Statistical analysis was performed using one-way ANOVA (*n* = 3; *p < 0.05, **p < 0.01, ***p < 0.001, compared with the control group; ^#^ *p* < 0.05, ^##^ *p* < 0.01; ^###^ *p* < 0.001, compared with the CDDP group). CBL: CDDP + BBR low dose; CBM: CDDP + BBR medium dose; CBH: CDDP + BBR high dose.

The levels of pro-inflammatory factors (NF-κB, NGF, IL-1*β*, IL-6, and TNF-*α*) in serum were decreased in a dose-dependent manner for 2 weeks and, after 4 weeks of treatment with BBR, the level of anti-inflammatory factor IL-10 was increased. There was no difference between wild type group and *TRPV1* ^*−/−*^ group (Figures 2A,2B, 6A,6B). These results suggested that serum inflammatory factors may be upstream factors that triggered intracellular signal transduction in DRG, so they were not affected by deletion of TRPV1.

### 3.7 Effect of Berberine on the NF-κB and JNK/p38 MAPK/ERK Pathways in Dorsal Root Ganglia of TRPV1 ^−/−^ Mice

**FIGURE 7:**
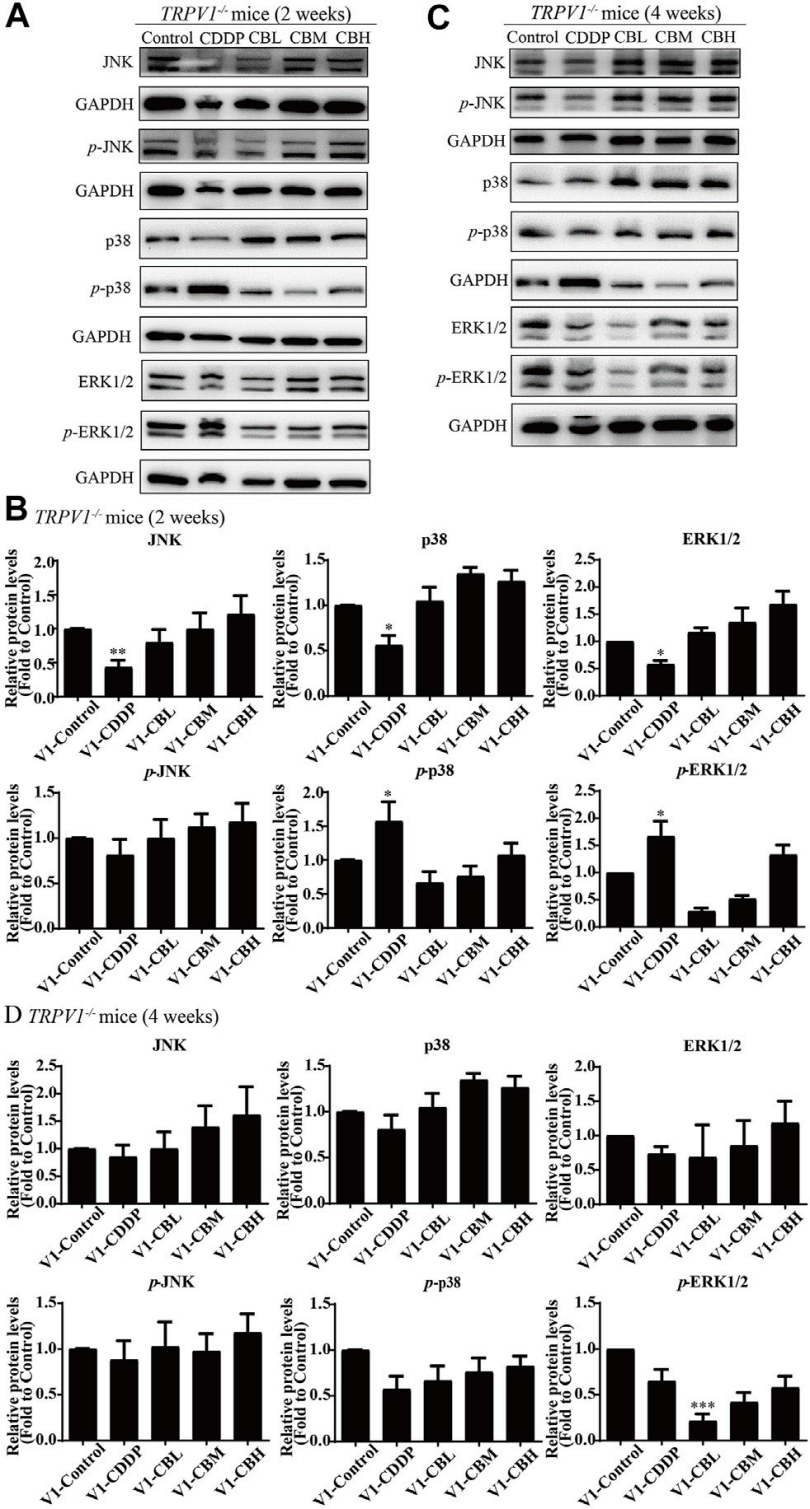
BBR regulates TRPV1-mediated MAPKs signaling pathway in mice. Western blot showed the levels of MAPKs inflammatory pathway protein in the DRGs of *TRPV1* ^*−/−*^ mice (*n* = 3). **(A,B)** After 2 weeks of treatment, BBR up-regulated the downregulation expression of JNK, p38, ERK1/2 induced by CDDP, and inhibited the phosphorylation of p38(*F* = 8.702, *p* = 0.0008) and ERK1/2 (*F* = 6.228, *p* = 0.0037) in *TRPV1* ^*−/−*^ mice. **(C,D)** There was no significant difference in MAPKs signaling pathway protein expression in each group of mice after 4 weeks of treatment. Statistical analysis was performed using one-way ANOVA (*p < 0.05, **p < 0.01, ***p < 0.001, compared with the control group; ^#^ *p* < 0.05, ^##^ *p* < 0.01; ^###^ *p* < 0.001, compared with the CDDP group). CBL: CDDP + BBR low dose; CBM: CDDP + BBR medium dose; CBH: CDDP + BBR high dose.

There was no difference in the protein expression of NF-κB in DRG of *TRPV1* ^*−/−*^ mice at the second week (Figures 6C,E,G,I). At the fourth week, NF-κB expression was increased in the CDDP group, and BBR was shown to relieve neurological symptoms by inhibiting NF-κB expression (Figures 6D,F,H,J). In our previous study, BBR inhibited the expression of NF-κB induced by CDDP after 2 weeks of treatment and promoted the expression of NF-κB after 4 weeks of treatment in WT mice (Figure 2). This indicated that NF-κB expression was completely blocked by *TRPV1* knockout at an early stage in CDDP animals (Figures 2C,E, 6C,E). However, even the *TRPV1* gene was knocked out, the level of NF-κB protein was still elevated in the late model of CDDP (Figures 3D,F, 6D,F), and BBR inhibited the high expression of NF-κB in late-stage CIPN. Moreover, the expression of JNK, p38, and ERK1/2 decreased and *p*-p38 and *p*-ERK1/2 increased in response to CDDP in DRG of *TRPV1* ^−/−^ mice. After 2 weeks of treatment, BBR maintained the expression of JNK, p38, and ERK1/2, and inhibited the phosphorylation of p38 and ERK1/2 in *TRPV1* ^−/−^ mice (Figures 7A,B). In contrast, BBR treatment promoted the phosphorylation of p38 and ERK1/2 in WT mice at 2 weeks (Figures 3A,B). This suggested that BBR may regulate JNK/p38 MAPK/ERK inflammatory signalling mediated by the *TRPV1* receptor in CIPN. At 4 weeks, there was no significant difference in MAPK signalling pathways proteins expression in *TRPV1* knockout mice before and after BBR treatment (Figures 7C,D).

### 3.8 Berberine Alleviates Cultured Dorsal Root Ganglia Neuron Damage Induced by CDDP

**FIGURE 8:**
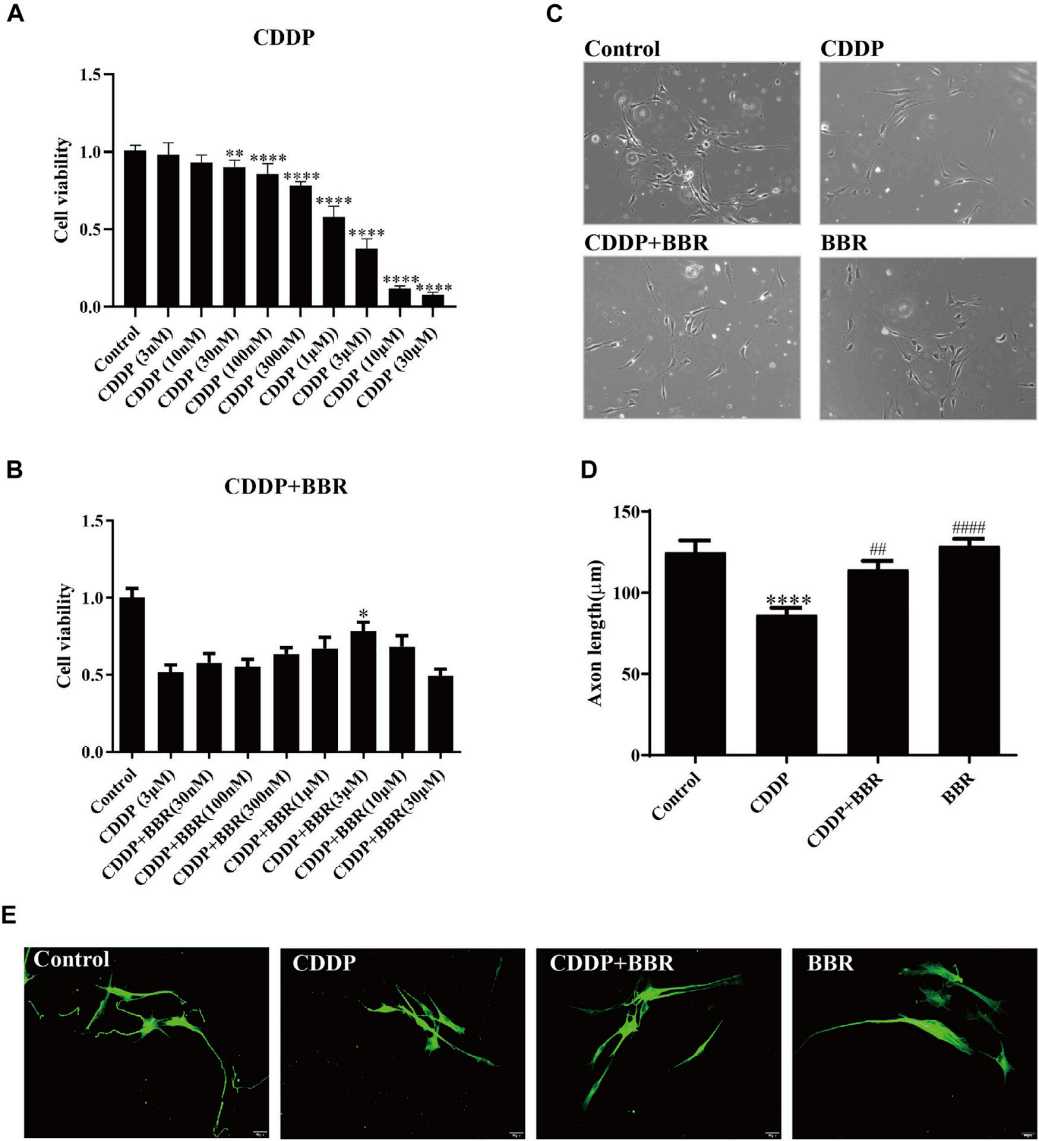
BBR protects DRG cells from damage induced by CDDP. **(A)** The MTT assay was used to measure the survival rate of neurons. DRG neurons were cultured with CDDP (3 µM) for 48 h, the cell viability was decreased to 40–50% (*n* = 6, *F* = 301.7, *p* < 0.0001). **(B)** An injury model of neurons cultured with CDDP (3 μM) was established, and then DRG was cultured with different concentration gradient BBR for 48 h. The results showed that BBR (3 μM) enhanced cell viability (*F* = 8.034, *p* < 0.0001). **(C)** Primary DRG neuronal cell were cultured with CDDP (3 µM). **(D)** CDDP (3 μM) contributes to the axon growth, whereas BBR (3 μM) co-incubated with CDDP protected normal axon growth in neurons or BBR (3 µM) for 48 h (*F* = 13.59, *p* < 0.0001). **(E)** Neuron specific nucleoprotein *β*III-tubulin staining was used to identify primary DRG cell axons. BBR (3 μM) attenuates the damage of CCDP to primary DRG cells. Statistical analysis was performed using one-way ANOVA (*p < 0.05, **p < 0.01, ***p < 0.001, ****p < 0.0001, compared with the control group; ^##^ *p* < 0.01, ^####^ *p* < 0.0001, compared with the CDDP group). CBL: CDDP + BBR low dose; CBM: CDDP + BBR medium dose; CBH: CDDP + BBR high dose.

To study whether BBR directly exerts anti-inflammatory effects on DRG, we cultured DRG neurons *in vitro* and observed changes in intracellular inflammatory signals. After incubation with 3 μM CDDP for 48 h, the neuronal cell viability was decreased to 40–50% (Figure 8A). 3 μM BBR treatment significantly enhanced cell viability (Figure 8B) compared with CDDP group using the MTT assay. Then, the morphology of DRG neurons was examined (Figure 8C). Measuring the axon length of neurons, 3 μM CDDP was found to cause axon degeneration, whereas 3 μM BBR co-incubated with CDDP protected normal axon growth in neurons (Figure 8D), which was consistent with the results of *β*III-tubulin immunofluorescence staining (Figure 8E).

### 3.9 Effect of Berberine on the NF-κB and JNK/p38 MAPK/ERK Pathways in Dorsal Root Ganglia Neurons

**FIGURE 9:**
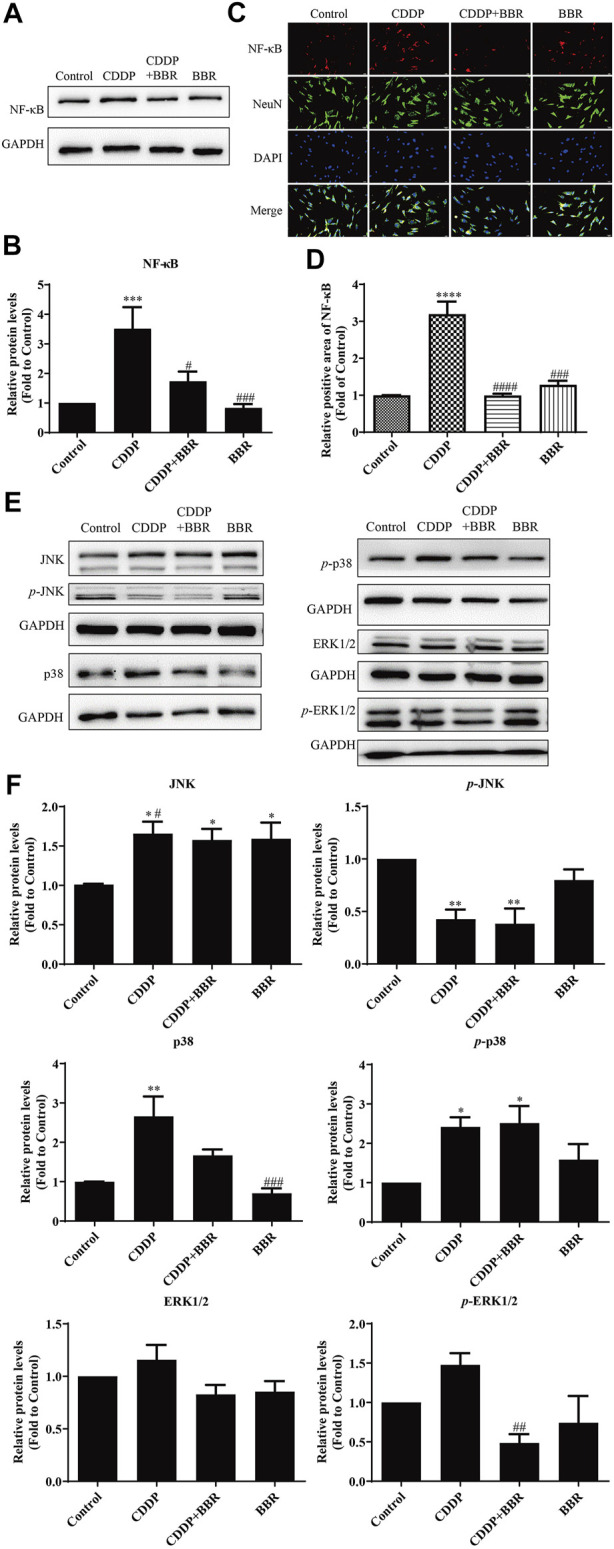
BBR inhibits the overexpression of NF-κB in DRG neurons induced by CDDP. **(A,B)** BBR inhibits the overexpression of NF-κB induced by CDDP (*F* = 9.178, *p* = 0.0002). **(C)** Immunofluorescence staining showed NF-κB relative abundance induced by CDDP and BBR stimulation in DRG cells NeuN labeled neurons. Scale bar = 20 μm. **(D)** The quantitative of immunofluorescence showed that 3 μM BBR alleviates the expression of NF- κB in DRG tissue (*F* = 43.49, *p* < 0.0001). **(E,F)** CDDP (3 μM, 48 h) increased the expression of JNK, p38, and *p*-p38, and decreased the expression of *p*-JNK, *p*-ERK1/2 and p38. Co-incubation with 3 μM BBR inhibited the expression of *p*-ERK1/2 (*F* = 4.764, *p* = 0.0207) and p38 (*F* = 10.3, *p* = 0.0005). Statistical analysis was performed using one-way ANOVA (*n* = 3; *p < 0.05, **p < 0.01, ***p < 0.001, ****p < 0.0001, compared with the control group; ^#^ *p* < 0.05, ^##^ *p* < 0.01, ^###^ *p* < 0.001, ^####^ *p* < 0.0001, compared with the CDDP group). CBL: CDDP + BBR low dose; CBM: CDDP + BBR medium dose; CBH: CDDP + BBR high dose.

Western blot and immunofluorescence staining were used to examine the expression of NF-κB 48 h after treatment with BBR. The results showed that CDDP caused overexpression of NF-κB in neurons, and BBR normalized the protein level (Figures 9A–D). Hence BBR may reduce the inflammatory damage of DRG neurons induced by CDDP by inhibiting NF-κB signalling. Furthermore, the expression of JNK, p38, and *p*-p38 increased and the expression of *p*-JNK decreased in DRG neurons incubated with 3 μM CDDP for 48 h. Co-incubation with 3 μM BBR inhibited the expression of *p*-ERK1/2 and p38 (Figures 9E,F). The results indicate that BBR can reduce damage to DRG neurons by regulating JNK/p38 MAPK/ERK inflammatory signalling *in vitro*.

## 4 Discussion

This study showed that a single dose of CDDP caused thermal hyperalgesia for 2 h in mice, and then the thermal hyperalgesia gradually disappeared in 1–2 h. Long-term administration of CDDP for 2 weeks can cause peripheral nerve pain abnormalities. BBR attenuated peripheral hyperalgesia induced by CDDP (single dose and long-term) in mice, and its improvement was similar to the duloxetine group. Experiments *in vitro* demonstrated that BBR can increase the activity of DRG neurons and reduce the damage of CDDP to cells. Previous studies have shown that BBR has neuroprotective effects, it can alleviate diabetic peripheral neuropathy (Yang wt al., 2020) and protect DRG neuronal cells from high glucose damage (Yerra et al., 2018). And other studies have suggested that BBR can reduce paclitaxel induced peripheral nerve injury (Singh et al., 2018). Our study indicates that BBR can be used to prevent and treat cisplatin induced peripheral neuropathy.

The success of CDDP injury model of primary cultured DRG neuronal cell can be identified by cell viability and morphological changes. Neuronal cell bodies and axons were identified respectively with neuron-specific nuclear protein NeuN and *β*-III tubulin (Guo et al., 2017). More and more studies have shown that oxidative stress in spinal dorsal root ganglion may underlie the pathogenesis of platinum induced peripheral neurotoxicity. The binding of platinum ions bind to DNA leads to mitochondrial dysfunction and DRG energy failure (Carozzi et al., 2010). *In vitro* and *in vivo* studies have shown that CDDP induced neuronal apoptosis in DRG was associated with changes in cyclin expression (Fischer et al., 2001; Mcdonald et al., 2005), and MAPK family members (ERK1/2, JNK, p38) are involved in peripheral nerve injury (Myers et al., 2003; Zielinska, 2003; Cavaletti et al., 2010; Belair, 2021). Platinum induced apoptosis of DRG neuronal is mediated by the early activation of p38 and ERK1/2 (Scuteri et al., 2009), whereas JNK/SAPK is involved in cell repair and protects neurons from platinum ion damage (Middlemas et al., 2003; Potapova et al., 1997). All of the above studies suggest that platinum ion induced peripheral neuropathy triggers oxidative stress and apoptosis of DRG neurons through the MAPK signalling pathways.

Previous studies have shown that BBR can protect neurons by anti-apoptotic, anti-oxidative, and anti-inflammatory effects through PI3K/Akt/Bcl-2, Akt/NF-κB, and MAPK signalling (Zhang et al., 2018). BBR have reduced neuronal damage both *in vivo* and *in vitro* by inhibiting inflammatory responses and reducing the production of inflammatory mediators rather than directly exerting anti-inflammatory effects on neuronal cells. BBR have inhibited the expression and activity of tissue factors induced by lipopolysaccharide, whereas NF-κB, AKT, and JNK/p38 MAPK/ERK pathways were downregulated (Gao et al., 2014). BBR have also exerted anti-inflammatory effects by inhibiting COX-2 (Feng et al., 2012). As a treatment for traumatic inflammation, BBR inhibited TAK1/JNK and TAK1/NF-κB signalling by reducing phosphorylation of JNK and NF-κB, and reducing the expression of inflammation factors IL-1*β*, IL-6, IL-8, TNF-*α*, and TGF-*β* to inhibit inflammation (Akhter et al., 1977; Zhang et al., 2014). In addition, BBR have blocked TLR4 receptors to decrease TLR4 mRNA and protein expression, attenuated NF-κB activity, reduced secretion of inflammatory factors IL-1 and IL-6, and preventd macrophage involvement in inflammatory response (Fu et al., 2015). BBR have significantly inhibited the MAPK pathways by blocking phosphorylation of p38 and JNK rather than phosphorylation of ERK1/2 (Feng et al., 2017). BBR have partially inhibited activation of TRPV1 by inhibiting inflammation and blocking the PKC pathway, thereby protecting peripheral neurons from damage caused by diabetes (Zan et al., 2017). Although these studies were not primarily directed at CIPN, they suggest that the mechanism by which BBR reduces CIPN nerve injury is associated with regulation of inflammatory signalling pathways.

Our study showed that BBR can inhibit the CDDP induced neuroinflammatory response by inhibiting TRPV1 and NF-κB protein overexpression, activating the JNK/p38 MAPK pathways, and promoting NF-κB expression in early-stage injury. In late-stage injury, BBR inhibited the expression of *p*-JNK, promoted NF-κB expression, and regulated the p38 MAPK/ERK signalling pathways. BBR dose-dependently decreased pro-inflammatory factors NF-κB, IL-1*α*, IL-1*β*, and IL-6 in the serum of mice. The increase in the anti-inflammatory factor IL-10 improved hyperalgesia symptoms in mice. To sum up, our results indicate that BBR can inhibit NF-κB protein expression by blocking TRPV1, decrease phosphorylation of p38 and ERK1/2, and ameliorate thermal hyperalgesia induced by CDDP.

The expression of TRPV1 in DRG is an important target for pain research. Studies have shown that upregulation of *TRPV1* and *TRPA1* mRNA was associated with thermal hyperalgesia and mechanical hypersensitivity in CDDP treated mice (Bölcskei et al., 2005; Ta et al., 2009). This suggests that TRPV1 plays an important role in signalling associated with thermal heat pain after CDDP induced neuronal injury. In this study, we compared CDDP induced thermal heat pain motility behaviour changes before and after *TRPV1* gene knockout in mice, and found that nerve fibres expressed thermal hyperalgesia induced by CDDP. After knocking out the *TRPV1* gene, the hyperalgesia response was controlled within 2 weeks, but the thermal heat pain threshold was still reduced 3 weeks after treatment. BBR reversed CDDP induced pain sensation throughout the course of the experiment. These results suggest that TRPV1 receptors are only involved in regulating CDDP induced hyperalgesia in the early stage of BBR therapy. Other mechanisms may be involved in the later stages of CIPN treatment.

We found that BBR decreased the levels of proinflammatory factors NF-κB, IL-1*α*, IL-1*β*, IL-6, and TNF-*α* in mice serum and increased the levels of anti-inflammatory factor IL-10 in a dose-dependent manner. It indicates that the anti-inflammatory effect of BBR is not only expressed in DRG, but also involved in regulating blood. Previous studies have shown that NGF acted as an inflammatory mediator, activating PKA, PKC, MAPK/ERK, and PI3K signalling through sensitising TRPV1 receptors, leading to thermal hyperalgesia (Bölcskei et al., 2005; Zan et al., 2017). NGF can also upregulate TRPV1 expression through p38 MAPK signalling. With the activation of TRPV1 channel, calcium influx increases, and the TRPV1 channel threshold is reduced significantly during inflammation (Davis et al., 2000; Brederson et al., 2013; Maeda and Ozaki, 2014; Taguchi, 2016). Although many studies have shown that platinum drugs can increase expression of TRPV1 in animals (Chen et al., 2011; Khasabova et al., 2012; Naziroğlu and Braidy, 2017), there are no reports on these changes (Lu et al., 2020) or even a reduction in *TRPV1* mRNA expression in DRG neurons *in vitro* (Ta et al., 2009). The role of TRPV1 in CIPN remains to be clarified.

## Data Availability

The original contributions presented in the study are included in the article/Supplementary Material, further inquiries can be directed to the corresponding author.

## References

[B2] Akhter M. H. Sabir M. Bhide N. K. (1977). Anti-Inflammatory Effect of Berberine in Rats Injected Locally with Cholera Toxin. Indian J. Med. Res. 65 (1), 133–141. 863481

[B3] Belair D. G. (2021). Investigation into the Role of ERK in Tyrosine Kinase Inhibitor-Induced Neuropathy. London: Oxford University Press. 10.1093/toxsci/kfab033 33749749

[B19] Brederson J. D. Kym P. R. Szallasi A. (2013). Targeting TRP Channels for Pain Relief. Eur. J. Pharmacol. Int. J. 716 (1–3), 61–76. 10.1016/j.ejphar.2013.03.003 23500195

[B4] Bölcskei K. Helyes Z. Szabó A. Sándor K. Elekes K. Németh J. (2005). Investigation of the Role of TRPV1 Receptors in Acute and Chronic Nociceptive Processes Using Gene-Deficient Mice. Pain. 117 (3), 368–376. 10.1016/j.pain.2005.06.024 16150543

[B5] Carozzi V. A. Chiorazzi A. Canta A. Meregalli C. Oggioni N. Cavaletti G. (2014). Chemotherapy-induced Peripheral Neurotoxicity in Immune-Deficient Mice: New Useful Ready-To-Use Animal Models. Exp. Neurol. 264 (2), 92–102. 10.1016/j.expneurol.2014.11.002 25450467

[B6] Carozzi V. A. Marmiroli P. Cavaletti G. (2010). The Role of Oxidative Stress and Anti-Oxidant Treatment in Platinum-Induced Peripheral Neurotoxicity. Curr. Cancer Drug Targets. 10 (7), 670–682. 10.2174/156800910793605820 20578989

[B7] Caterina M. J. Leffler A. Malmberg A. B. Martin W. J. Trafton J. Petersen-Zeitz K. R. (2000). Impaired Nociception and Pain Sensation in Mice Lacking the Capsaicin Receptor. Science. 288 (5464), 306–313. 10.1126/science.288.5464.306 10764638

[B8] Caterina M. J. Schumacher M. A. Tominaga M. Rosen T. A. Levine J. D. Julius D. (1997). The Capsaicin Receptor: a Heat-Activated Ion Channel in the Pain Pathway. Nature. 389 (6653), 816–824. 10.1038/39807 9349813

[B9] Cavaletti G. Miloso M. Nicolini G. Scuteri A. Tredici G. (2010). Emerging Role of Mitogen-Activated Protein Kinases in Peripheral Neuropathies. J. Peripher. Nerv Syst. 12 (3), 175–194. 10.1111/j.1529-8027.2007.00138.x 17868245

[B10] Chen Y. Yang C. Wang Z. J. (2011). Proteinase-Activated Receptor 2 Sensitizes Transient Receptor Potential Vanilloid 1, Transient Receptor Potential Vanilloid 4, and Transient Receptor Potential Ankyrin 1 in Paclitaxel-Induced Neuropathic Pain. Neuroscience. 193 (1), 440–451. 10.1016/j.neuroscience.2011.06.085 21763756

[B11] Davis J. B. Gray J. Gunthorpe M. J. Hatcher J. P. Davey P. T. Overend P. (2000). Vanilloid Receptor-1 Is Essential for Inflammatory Thermal Hyperalgesia. Nature. 405 (6783), 183–187. 10.1038/35012076 10821274

[B1] Feng A. W. Gao W. Zhou G. R. Yu R. Li N. Huang X. L. (2012). Berberine Ameliorates COX-2 Expression in Rat Small Intestinal Mucosa Partially Through PPARγ Pathway During Acute Endotoxemia. Int. Immunopharmacology. 12 (1), 182–188. 10.1016/j.intimp.2011.11.009 22155099

[B12] Feng M. Kong S. Z. Wang Z. X. He K. Zou Z. Y. Hu Y. R. (2017). The Protective Effect of Coptisine on Experimental Atherosclerosis ApoE-/- Mice Is Mediated by MAPK/NF-κB-Dependent Pathway. Biomed. Pharmacother. 93, 721–729. 10.1016/j.biopha.2017.07.002 28700976

[B13] Fischer S. J. McDonald E. S. Gross L. Windebank A. J. (2001). Alterations in Cell Cycle Regulation Underlie Cisplatin Induced Apoptosis of Dorsal Root Ganglion Neurons *In Vivo* . Neurobiol. Dis. 8 (6), 1027–1035. 10.1006/nbdi.2001.0426 11741398

[B14] Fu K. Lv X. Li W. Wang Y. Li H. Tian W. (2015). Berberine Hydrochloride Attenuates Lipopolysaccharide-Induced Endometritis in Mice by Suppressing Activation of NF-Κb Signal Pathway. Int. Immunopharmacol. 24 (1), 128–132. 10.1016/j.intimp.2014.11.002 25479718

[B41] Fukuda Y. Li Y. Segal R. A. (2017). A Mechanistic Understanding of Axon Degeneration in Chemotherapy-Induced Peripheral Neuropathy. Front. Neurosci. 11, 481. 10.3389/fnins.2017.00481 28912674 PMC5583221

[B15] Gao M. Y. Chen L. Yang L. Yu X. Kou J. P. Yu B. Y. (2014). Berberine Inhibits LPS-Induced TF Procoagulant Activity and Expression through NF-κB/p65, Akt and MAPK Pathway in THP-1 Cells. Pharmacol. Rep. 66 (3), 480–484. 10.1016/j.pharep.2013.12.004 24905527

[B16] Guo L. Hamre J. Eldridge S. Behrsing H. P. Cutuli F. M. Mussio J. (2017). Multiparametric Image Analysis of Rat Dorsal Root Ganglion Cultures to Evaluate Peripheral Neuropathy-Inducing Chemotherapeutics. Toxicol. Sci. 156 (1), 275–288. 10.1093/toxsci/kfw254 28115644 PMC5837782

[B18] Ibrahim E. Y. Ehrlich B. E. (2020). Prevention of Chemotherapy-Induced Peripheral Neuropathy: A Review of Recent Findings. Crit. Rev. Oncol. Hematol. 145, 102831. 10.1016/j.critrevonc.2019.102831 31783290 PMC6982645

[B20] Khasabova I. A. Khasabov S. Paz J. Harding-Rose C. Simone D. A. Seybold V. S. (2012). Cannabinoid Type-1 Receptor Reduces Pain and Neurotoxicity Produced by Chemotherapy. J. Neurosci. 32 (20), 7091–7101. 10.1523/JNEUROSCI.0403-12.2012 22593077 PMC3366638

[B21] Livak K. J. Schmittgen T. D. (2001). Analysis of Relative Gene Expression Data Using Real-Time Quantitative PCR and the 2(-Delta Delta C(T)) Method. Methods. 25 (4), 402–408. 10.1006/meth.2001.1262 11846609

[B22] Lu Y. Zhang P. Zhang Q. Yang C. Qian Y. Suo J. (2020). Duloxetine Attenuates Paclitaxel-Induced Peripheral Nerve Injury by Inhibiting P53-Related Pathways. J. Pharmacol. Exp. Ther. 373 (3), 453265082–453265462. 10.1124/jpet.120.265082 32238452

[B23] Maeda T. Ozaki M. (2014). Study on Novel Mechanism Underlying Analgesia Targeting TRPV1. Yakugaku Zasshi. 134 (3), 373–378. 10.1248/yakushi.13-00236-1 24584018

[B24] Mcdonald E. S. Randon K. R. Knight A. Windebank A. J. (2005). Cisplatin Preferentially Binds to DNA in Dorsal Root Ganglion Neurons *In Vitro* and *In Vivo*: a Potential Mechanism for Neurotoxicity. Neurobiol. Dis. 18 (2), 305–313. 10.1016/j.nbd.2004.09.013 15686959

[B25] Middlemas A. Delcroix J. D. Sayers N. M. Tomlinson D. R. Fernyhough P. (2003). Enhanced Activation of Axonally Transported Stress-Activated Protein Kinases in Peripheral Nerve in Diabetic Neuropathy Is Prevented by Neurotrophin-3. Brain. 126 (7), 1671–1682. 10.1093/brain/awg150 12805110

[B27] Myers R. R. Sekiguchi Y. Kikuchi S. Scott B. Medicherla S. Protter A. (2003). Inhibition of P38 MAP Kinase Activity Enhances Axonal Regeneration. Exp. Neurol. 184 (2), 606–614. 10.1016/S0014-4886(03)00297-8 14769353

[B26] Naziroğlu M. Braidy N. (2017). Thermo-Sensitive TRP Channels: Novel Targets for Treating Chemotherapy-Induced Peripheral Pain. Front. Physiol. 8, 1040. 10.3389/fphys.2017.01040 29326595 PMC5733463

[B28] Ni W. Zheng X. Hu L. Kong C. Xu Q. (2021). Preventing Oxaliplatin-Induced Neuropathic Pain: Using Berberine to Inhibit the Activation of NF-Κb and Release of Pro-Inflammatory Cytokines in Dorsal Root Ganglions in Rats. Exp. Ther. Med. 21 (2), 135. 10.3892/etm.2020.9567 33376517 PMC7751485

[B29] Potapova O. Haghighi A. Bost F. Liu C. Birrer M. J. Gjerset R. (1997). The Jun Kinase/Stress-Activated Protein Kinase Pathway Functions to Regulate DNA Repair and Inhibition of the Pathway Sensitizes Tumor Cells to Cisplatin. J. Biol. Chem. 272 (22), 14041–14044. 10.1074/jbc.272.22.14041 9162025

[B31] Quasthoff S. Hartung H. P. (2002). Chemotherapy-induced Peripheral Neuropathy. J. Neurol. 249 (1), 9–17. 10.1007/pl00007853 11954874

[B32] Scuteri A. Galimberti A. Maggioni D. Ravasi M. Pasini S. Nicolini G. (2009). Role of MAPKs in Platinum-Induced Neuronal Apoptosis. Neurotoxicology. 30 (2), 312–319. 10.1016/j.neuro.2009.01.003 19428505

[B33] Singh J. Saha L. Singh N. Kumari P. Bhatia A. Chakrabarti A. (2019). Study of Nuclear Factorerythroid Related Factoractivator, Berberine, in Paclitaxel Induced Peripheral Neuropathy Pain Model in Rats. J. Pharm. Pharmacol. 71 (5), 797–805. 10.1111/jphp.13047 30536411

[B34] Staff N. P. Grisold A. Grisold W. Windebank A. J. (2017). Chemotherapy-Induced Peripheral Neuropathy: A Current Review. Ann. Neurol. 81 (6), 772–781. 10.1002/ana.24951 28486769 PMC5656281

[B35] Ta L. E. Bieber A. J. Carlton S. M. Loprinzi C. L. Low P. A. Windebank A. J. (2010). Transient Receptor Potential Vanilloid 1 Is Essential for Cisplatin-Induced Heat Hyperalgesia in Mice. Mol. Pain. 6 (1), 15. 10.1186/1744-8069-6-15 20205720 PMC2848188

[B36] Ta L. E. Low P. A. Windebank A. J. (2009). Mice with Cisplatin and Oxaliplatin-Induced Painful Neuropathy Develop Distinct Early Responses to thermal Stimuli. Mol. Pain. 5 (1), 9. 10.1186/1744-8069-5-9 19245717 PMC2655284

[B37] Taguchi K. (2016). Role of Transient Receptor Potential Channels in Paclitaxel- and Oxaliplatin-Induced Peripheral Neuropathy. Yakugaku Zasshi. 136 (2), 287–296. 10.1248/yakushi.15-00214 26831807

[B38] Wu J. Zhang H. Hu B. Yang L. Wang P. Wang F. (2016). Coptisine from Coptis Chinensis Inhibits Production of Inflammatory Mediators in Lipopolysaccharide-Stimulated RAW 264.7 Murine Macrophage Cells. Eur. J. Pharmacol. 780, 106–114. 10.1016/j.ejphar.2016.03.037 27018392

[B30] Xu Q. Zhang X. M. Duan K. Z. Gu X. Y. Han M. Liu B. L. (2013). Peripheral TGF-ß 1 Signaling Is a Critical Event in Bone Cancer-Induced Hyperalgesia in Rodents. J. Neurosci. 33 (49), 19099–19111. 10.1523/JNEUROSCI.4852-12.2013 24305807 PMC6618781

[B39] Yang S. Yu Z. Sun W. Jiang C. Ba X. Zhou Q. (2020). The Antiviral Alkaloid Berberine Ameliorates Neuropathic Pain in Rats with Peripheral Nerve Injury. Acta Neurol. Belgica. 120 (3), 557–567. 10.1007/s13760-018-1006-9 30168114

[B40] Yerra V. G. Kalvala A. K. Sherkhane B. Areti A. Kumar A. (2018). Adenosine Monophosphate-Activated Protein Kinase Modulation by Berberine Attenuates Mitochondrial Deficits and Redox Imbalance in Experimental Diabetic Neuropathy. Neuropharmacology. 131, 256–270. 10.1016/j.neuropharm.2017.12.029 29273519

[B42] Zan Y. Kuai C. X. Qiu Z. X. Huang F. (2017). Berberine Ameliorates Diabetic Neuropathy: TRPV1 Modulation by PKC Pathway. Am. J. Chin. Med. 45, 1709–1723. 10.1142/S0192415X17500926 29121795

[B43] Zhang Y. Li X. Zhang Q. Li J. Ju J. Du N. (2014). Berberine Hydrochloride Prevents Postsurgery Intestinal Adhesion and Inflammation in Rats. J. Pharmacol. Exp. Ther. 349 (3), 417–426. 10.1124/jpet.114.212795 24676878

[B17] Zhang H. N. Sun Y. J. He H. Q. Li H. Y. Xie Q. L. Liu Z. M. (2018). Berberine Promotes Nerve Regeneration Through IGFR-Mediated JNK-AKT Signal Pathway. Mol. Med. Rep. 18 (6), 5030–5036. 10.3892/mmr.2018.9508 30272344 PMC6236264

[B44] Zielinska M. (2003). Role of N-Methyl-D-Aspartate Receptors in the Neuroprotective Activation of Extracellular Signal-Regulated Kinase 1/2 by Cisplatin. J Biol Chem. 278, 43663. 10.1074/jbc.M301554200 12930843

